# Comprehensive analysis of human protein N-termini enables assessment of various protein forms

**DOI:** 10.1038/s41598-017-06314-9

**Published:** 2017-07-26

**Authors:** Jeonghun Yeom, Shinyeong Ju, YunJin Choi, Eunok Paek, Cheolju Lee

**Affiliations:** 1 Center for Theragnosis, Korea Institute of Science and Technology, Seoul, 02792 Republic of Korea; 2 Department of Biological Chemistry, Korea University of Science and Technology, Daejeon, 34113 Republic of Korea; 3 Department of Life Science and Research Center for Natural Sciences, Hanyang University, Seoul, 04763 Republic of Korea; 4 Department of Computer Science and Engineering, Hanyang University, Seoul, 04763 Republic of Korea

## Abstract

Various forms of protein (proteoforms) are generated by genetic variations, alternative splicing, alternative translation initiation, co- or post-translational modification and proteolysis. Different proteoforms are in part discovered by characterizing their N-terminal sequences. Here, we introduce an N-terminal-peptide-enrichment method, Nrich. Filter-aided negative selection formed the basis for the use of two N-blocking reagents and two endoproteases in this method. We identified 6,525 acetylated (or partially acetylated) and 6,570 free protein N-termini arising from 5,727 proteins in HEK293T human cells. The protein N-termini included translation initiation sites annotated in the UniProtKB database, putative alternative translational initiation sites, and N-terminal sites exposed after signal/transit/pro-peptide removal or unknown processing, revealing various proteoforms in cells. In addition, 46 novel protein N-termini were identified in 5′ untranslated region (UTR) sequence with pseudo start codons. Our data showing the observation of N-terminal sequences of mature proteins constitutes a useful resource that may provide information for a better understanding of various proteoforms in cells.

## Introduction

The diversity of protein from a single gene is generated by several mechanisms, including co- or post-translational modification, alternative splicing and alternative translation initiation. These processes affect function, localization, or stability of the protein. In particular, there may be a significant disparity between N-terminal site predicted at the gene level and the N-terminal status of the protein product. For example, the half-life of a protein is affected by N-terminal modification, as described by the N-end rule^1–3^. The roles of Nα-acetylation are reported not only to protect proteins from degradation by the ubiquitin-proteasome system, but also to act as a general degradation signal^4, 5^. Protein translocation to the endoplasmic reticulum starts with recognition of the signal peptide present at the N-terminus of the protein by a signal-recognition particle. Cleavage of the signal peptide generates a new N-terminus. The isoforms arising from alternative splicing, promoter and translation initiation site (TIS) can also generate a new N-terminal end that influences cell signaling pathways, cell death, and disease^6^. Thus, analysis of protein N-termini can reveal information about protein stability, localization, cleavage sites of proteases, and translation initiation sites. Recently, a mass-spectrometric analysis of human proteomes revealed proteins expressed from genes corresponding to approximately 84% of the total annotated protein coding genes in human^7^. However, such a comprehensive analysis reported only 4,105 annotated N-termini, which emphasizes the need for specialized N-terminal enrichment methods.

The approaches for identification of the N-termini of proteins are based on bottom-up proteomics and have been developed as positive or negative selection methods by which protein N-terminal peptides are isolated from other digested internal peptides. These approaches have recently been comprehensively reviewed^8, 9^. The basic process of these approaches can be divided into two main steps. The first step is a process to block the α-amine groups at the protein level to distinguish between N-terminal and internal peptides. The methods for labeling of the α-amine of proteins include enzymatic biotinylation^10^, chemical biotinylation^11^, iTRAQ (isobaric Tags for Relative and Absolute Quantification) labeling^12^, trideutero-acetylation^13^ and dimethylation^14^. Digestion with an endoprotease such as trypsin generates internal peptides with free α-amines, except for labeled N-terminal peptides. Here, a distinction is made between positive and negative selection depending on how to distinguish internal peptides. The positive selection method is that N-terminal peptides are enriched by affinity interaction, whereas the internal peptide in negative selection method is depleted with an amine-reactive agent such as a polyglycerol aldehyde polymer^15^. The major limitation of positive- selection methods is only detecting endogenous free protein N-termini, because acetylated (or otherwise modified) N-termini do not react with the affinity label. However, the negative selection can identify both naturally modified and free N-termini, thus the complexity of proteome is increased compared to positive selection. Well-established negative selection method can get more information of N-termini than positive selection. The two most widely reported negative selection methods are COmbined FRActional DIagonal Chromatography (COFRADIC)^13^ and terminal amine isotope labeling of substrates (TAILS)^15^. Recently, a study using COFRADIC found an average 1,452 annotated translation initiation sites per human cell line^16^. Another study using TAILS identified 7,094 protein N-termini from 3,485 proteins in human dental pulp^17^. There was also a report that found ~7,900 protein N-termini in human B cells by TAILS method without peptide prefractionation^18^. Although these approaches for the identification of protein N-termini have been successfully applied to many samples measuring protein turnover, determining translation initiation sites and confirming protein degradation, information of N-terminus for many proteins is still missing.

The purpose of the present study is to analyse N-terminal protein modification and to suggest a new approach for identification of protein N-termini based on negative selection. Generally, N-terminal-enrichment methods based on negative selection are carried out employing in-solution digestion. Experimenting with this digestion method, carry-over of N-blocking reagents in the next digestion step may affect subsequent analysis. In this paper, we have developed a negative selection method combined with filter-aided sample preparation (FASP)^19^. We term the method as N-terminal peptides enrichment on the filter (Nrich). The Nrich method was performed with two different N-blocking reagents and two endoproteases. As a result, we obtained 2,789 annotated translation initiation sites, 203 signal/transit and propeptide removal sites, 495 putative alternative translation initiation sites (aTIS) and 9,608 protein N-termini of hitherto unknown causes. The method we have developed utilizes multiple alternative materials, thus enabling larger coverage of N-terminal proteoforms in cells compared to single experiment. Our study proved that a certain protein(s) exist in various isoforms or multiple statuses in cells. The identification of N-terminome might provide a better understanding of various proteoforms in cells.

## Results and Discussion

### Deep-down enrichment of N-terminal peptides

**Figure 1:**
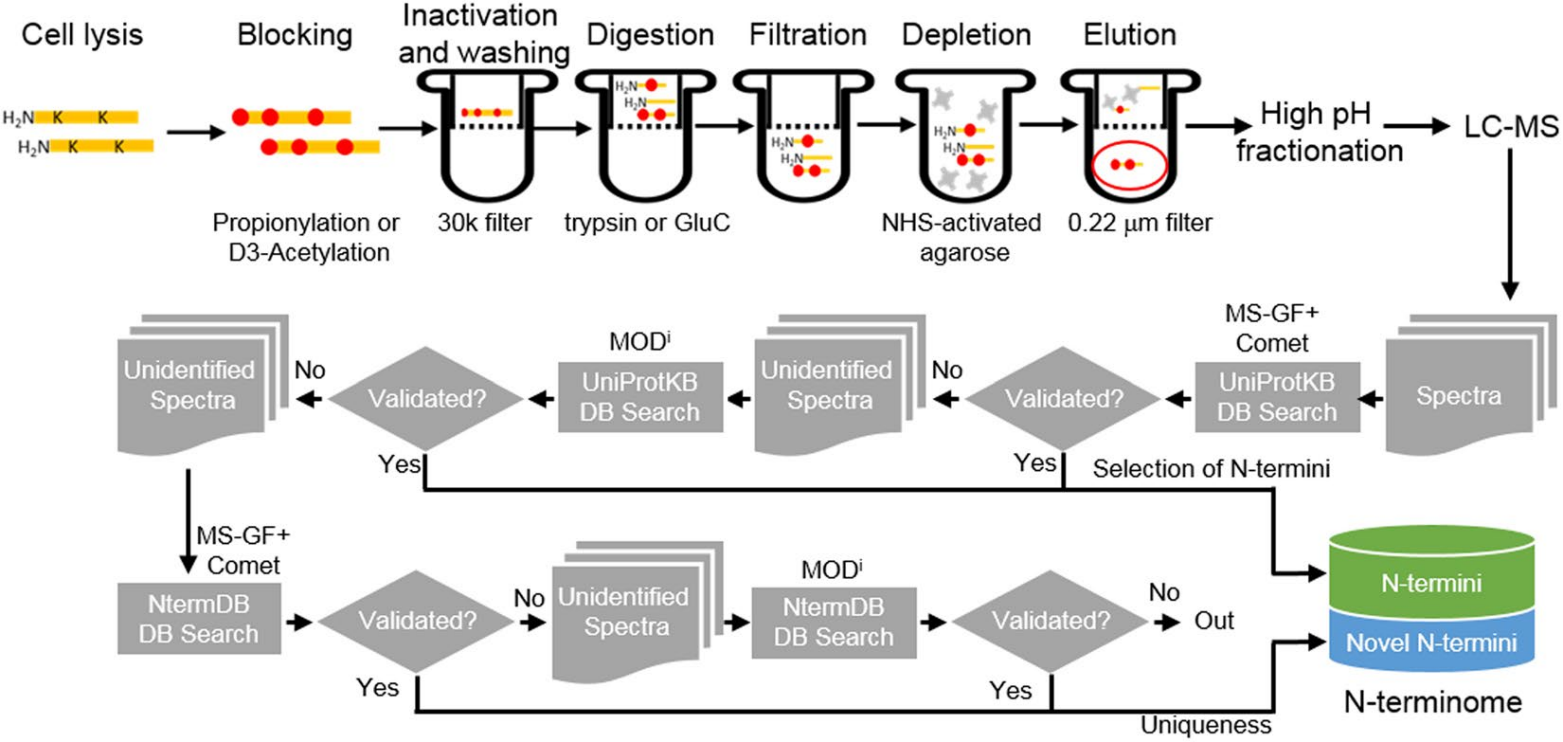
Outline of the Nrich method and the N-terminome discovery scheme. Proteins are labeled by D6-acetic anhydride or propionic anhydride to distinguish endogenous N-terminal acetylation from artificial N-terminal acetylation/propionylation. Followed by Filter Aided Sample Preparation (FASP) and digestion with trypsin or GluC endoprotease, internal peptides are depleted by using amine-reactive NHS beads. The enriched N-terminal peptides (red circle) are divided into 6 fractions by high pH reversed-phase fractionation. Then, all 6 fractions were subjected to LC-MS/MS analysis. The tandem MS spectra are initially searched against UniProtKB database with MS-GF+ and Comet search engines. Unidentified spectra are then selected to search for more diverse modifications using modification-specific search engine MOD^i^. Unidentified spectra following a UniProtKB database search combined with exploration of the three search engines were then put into the same search workflow after replacing the conventional database with a customized novel database, NtermDB. All the identifications on UniProtKB database were named “N-termini”, while the novel identifications on NtermDB were named “Novel N-termini.”

In order to characterize the status of proteins in cells, we performed “deep-down” N-terminal peptide enrichment (Nrich) in HEK293T cell line based on a negative selection method (Fig. 1, Methods). Nrich consisted of three major experimental steps: 1) the first step was to distinguish between endogenous Nα -acetylated and endogenous free N-termini. This was done by blocking α and ε primary amines of proteins with propionic anhydride (PA) or D6-acetic anhydride (D6). 2) Amine-blocked proteins were digested with trypsin or GluC-endoprotease using FASP methods^19^ for N-blocking reagent removal and buffer exchange. 3) Newly-generated internal peptides containing free α-amine were removed with a N-hydroxysuccinimide (NHS)-activated agarose resin. As a result, the peptides of the flow-through fraction in trypsin experiments were expected to have an ArgC-like digestion pattern because of the propionylation or trideuteroacetylation of the ε-amine of lysine. As expected, there was enrichment of endogenous Nα -acetylated peptides (Nt-acetylated N-termini) and *in vitro* Nα-propionylated or Nα-D3-acetylated peptides (free N-termini). The flow-through of NHS-agarose was separated by high-pH reversed-phase fractionation before LC-MS/MS. Mass spectral data were searched against the UniProtKB database using MS-GF+ and Comet followed by validation with percolator. Subsequently, the unidentified spectra from two search engines were re-analyzed by the MOD^i^ algorithm (Fig. 1). We used all the peptides found in any one search engine.

**Figure 2:**
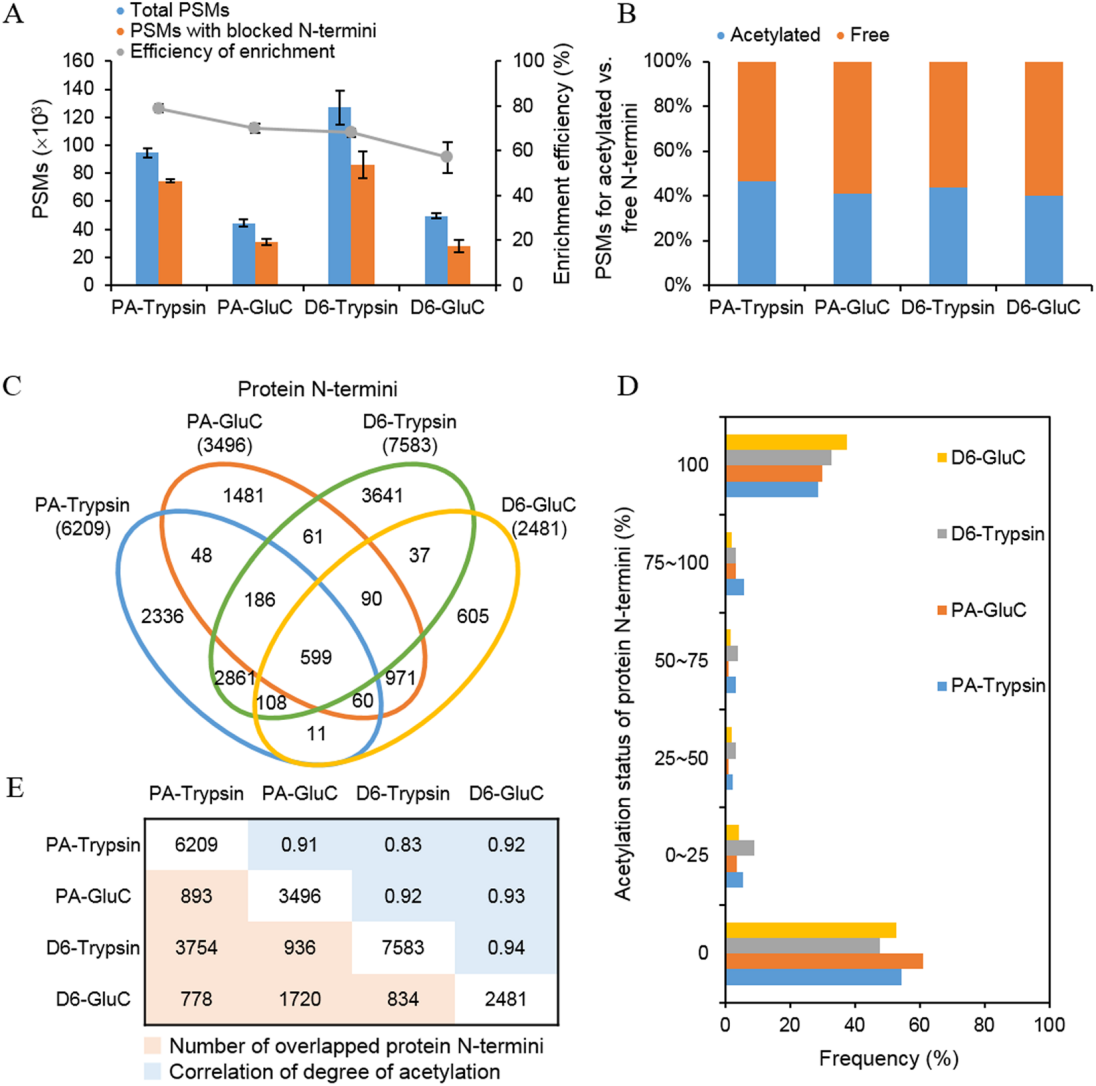
Properties of discovered N-terminome. (**A**) Number of PSMs identified on each set of Nrich experiment. In all cases, the trypsin-treated N-terminome have more PSMs than GluC-treated N-terminome. In the case of the N-blocking reagents, propionic anhydride (PA) showed higher efficiency by more than 10% compared to D6-acetic anhydride (D6). However, in the aspect of PSM counts, D6 treated samples had higher values than those of PA-treated samples. (**B**) Proportions of PSMs for endogenously acetylated N-termini (blue) and endogenously free N-termini (orange). About 44% of PSMs corresponded to acetylated N-termini. (**C**) Venn diagram of discovered protein N-termini according to different experimental setup. (**D**) Acetylation status of the discovered protein N-termini. The degree of acetylation was calculated based on the number of PSMs. (**E**) Protein N-termini discovered commonly between each pair of experimental setups and the correlation of degree of acetylation.

The enrichment efficacy for N-terminally blocked peptides was observed to be 79% (±2) in PA-Trypsin, 70% (±2) in PA-GluC, 68% (±2) in D6-Trypsin and 57% (±7) in D6-GluC. Peptide spectrum matches (PSMs) for N-terminally blocked peptides were counted in average as 74,456 in PA-Trypsin, 31,053 in PA-GluC, 86,115 in D6-Trypsin and 28,257 in D6-GluC at the false discovery rate (FDR) ≤0.01 (Fig. 2A). The number of identified N-termini varied depending on the nature of the N-blocking reagents and endoproteases, although it was reproducible for each experimental setup. The identified N-termini consisted of Nt-acetylated and free N-termini. Although Nrich incorporated a step for removal and inactivation of PA or D6 before endoprotease-digestion, we found PA- or D6-blocked internal peptides having protease-specific sites at both ends. Such peptides may have been generated during digestion by reagent traces (carried over from a prior step). Therefore, PA- or D6-labeled peptides with protease-specific sites were excluded from the final list except for the peptides found simultaneously in both trypsin and GluC experiments. Finally, we obtained 6,209 protein N-termini in PA-Trypsin, 3,496 in PA-GluC, 7,583 in D6-Trypsin and 2,481 in D6-GluC. We observed the almost equal amount of Nt-acetylated (44%) and Free (56%) N-termini in all experiments (Fig. 2B). Our method could enrich N-terminal peptides with similar efficacies regardless of N-blocking reagents or endoproteases. This observation is quite similar to results obtained by TAILS^17, 20^. Notably, our comprehensive analysis, using two N-blocking reagents and two endoproteases, greatly increased the coverage of the N-terminome. A total of 13,095 protein N-termini were identified as a sum in our study. Of these products, 62% were found exclusively by a single method (Fig. 2C). All four different methods were performed in biological and technical triplicates, and coefficients of variation values of the N-termini number were within 20%. Compared with the most frequently detected method, D6-Trypsin, the total number of all identified protein N-termini increased by 73%. Overall, the number of all protein N-termini, found by the four methods, increased by about 60% compared to a single experiment. In addition, we calculated the degree of acetylation for each of protein N-termini based on the number of PSMs (Fig. 2D). Most of the protein N-termini were identified as acetylated or free, and protein N-termini of partial acetylation were rarely found. Furthermore, the correlation of degree of acetylation between each different method was significantly high (0.83~0.94, Fig. 2E). These results lead us to the conclusion that the in cells status of protein N-termini can be determined irrespective of the nature of N-blocking reagents and endoproteases. However, the coverage of N-terminome can be significantly increased by combining the results from different enrichment methods.

### Classification of the N-terminome

**Figure 3:**
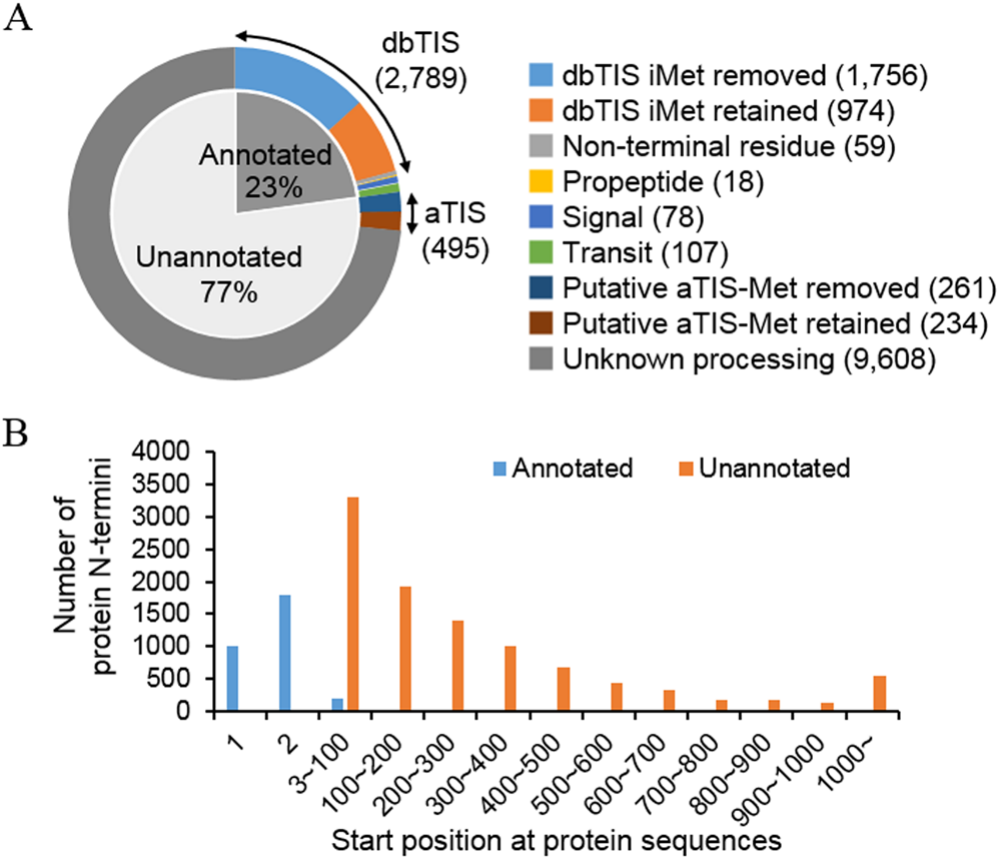
Classification of discovered N-termini and their positions along the protein sequences. (**A**) Classification of protein N-termini. dbTIS: UniProtKB-annotated translation initiation site; Non-terminal residue: protein N-termini starting with the first, but not methionine residue in the UniProtKB database; Propeptide/Signal/Transit: protein N-termini arising after removal of pro-, signal-, or transit-peptide; putative aTIS: putative alternative translation initiation site. (**B**) A number of protein N-termini identified according to their positions along the protein sequences.

The N-termini identified by Nrich originated from two different type of protein N-termini, e.g., Nt-acetylated and free N-termini (Nα-propionylated or Nα-D3-acetylated). We identified 6,525 acetylated (or partially acetylated) and 6,570 free N-termini. The N-terminome data can be classified based on their location, along with the corresponding protein sequences deposited in the protein database^16, 17, 20, 21^. Based on previous studies, we first divided the 13,095 N-termini of 5,727 proteins into 2,992 annotated (23%) and 10,103 unannotated (77%) protein N-termini (Fig. 3A). The annotated N-termini included UniProtKB-annotated translation initiation sites (dbTIS) and protein N-termini at sites after signal or transit peptide removal by post-translational processes (Supplementary Table S1). The dbTIS could be further sub-divided into protein N-termini, starting with initiator Met (iMet retained) and protein N-termini starting at the second residue without an initiator Met generated by co-translational modification (iMet removed), or those belonging to the ‘non-terminal residue category’ i.e., for those protein sequences in the UniProtKB database that do not start with methionine. Ninety-five percent of dbTIS originated from canonical protein sequences and 5% from isoform proteins. Generally, protein isoforms are produced by alternative splicing or alternative translation initiation. An interesting example is the identification of the acetylated N-terminal peptide of GSR (Glutathione reductase, P00390-2). The isoform is missing the first 43 residues of its canonical sequence. According to UniProt annotation, the subcellular location of the isoform (cytoplasm) is different from that of its canonical form (mitochondria). Protein N-termini of dbTIS category were compared with the ‘Terminus’ algorism^22^. As a result, the state of 74% protein N-termini was found to be the same as that predicted by the Terminus (Supplementary Table S1).

The unannotated protein N-termini accounted for the majority of our N-terminome data. Their positions along the corresponding protein sequences were greater than two (Supplementary Table S2). However, the signal peptides or other propeptides removed during protein maturation are not known (Fig. 3B). There are some hints that such unannotated N-termini might be interpreted as suggesting the possibility of aTISs or cleavage sites of proteolytic events. For example, we may regard 495 unannotated N-termini as aTIS because these N-termini were observed either starting with or immediately after internal methionine and 45% of these were acetylated. In addition, 412 N-termini were mapped to proteoforms found in such databases as Degrabase^23^, TopFIND database^24, 25^ and Proteoform Repository (http://repository.topdownproteomics.org/). Although it requires further examination, our interpretation is quite plausible since our data share many similarities with other previous investigations^16, 17, 20, 23, 26–28^.

### Features of annotated protein N-termini

**Figure 4:**
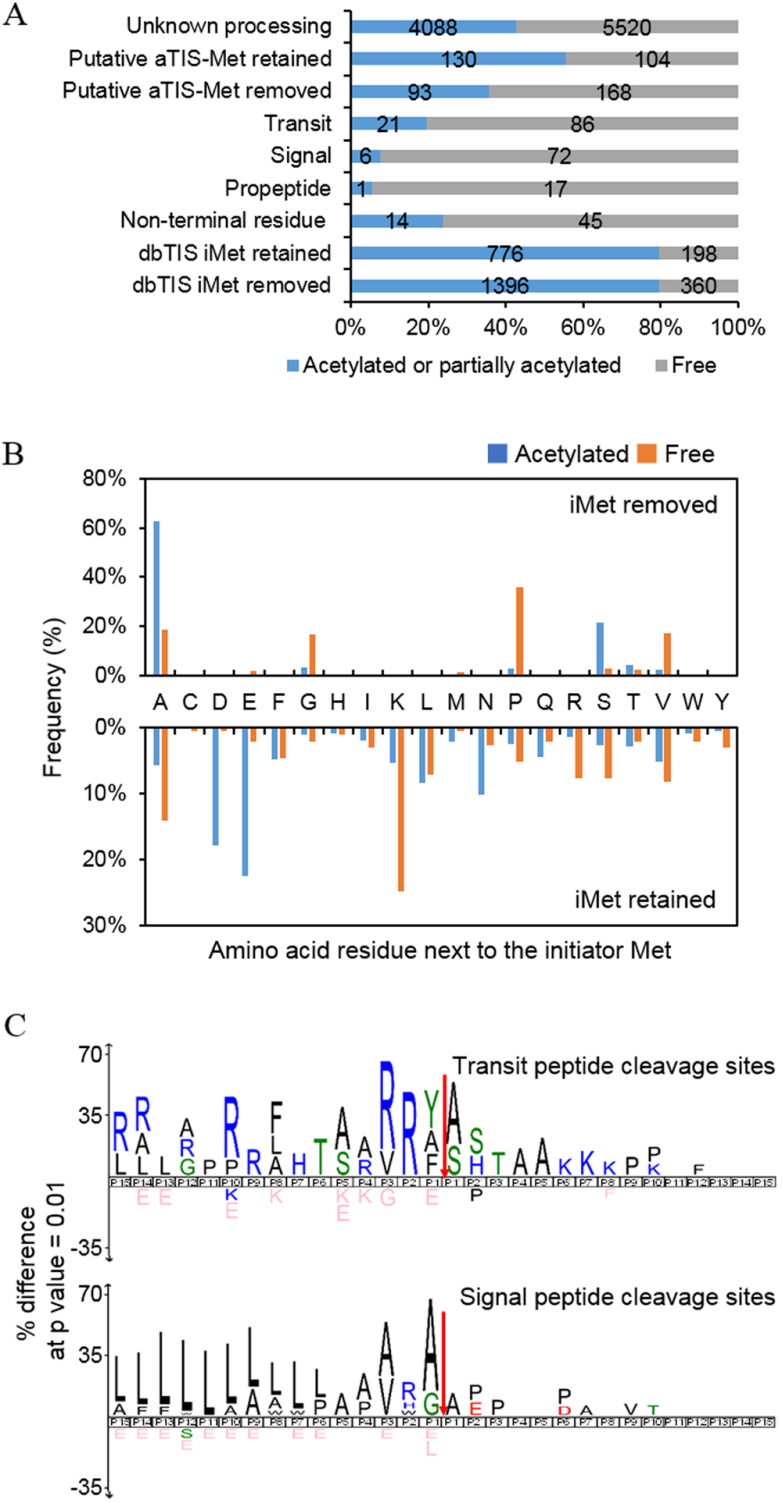
Features of protein N-termini. (**A**) Distribution of acetylated N-termini and free N-termini according to the type of N-termini. The data are presented in percentage values, and the exact numbers of protein-N-termini are denoted within bars. (**B**) The amino acid frequency at the second residue of acetylated or free protein N-termini. ‘The second residue’ means the residue next to the initiator methionine. (**C**) The amino acid frequency at the flanking region of signal peptides and transit peptides. The protein sequence logos were generated using the iceLogo software package with correction for natural amino acid abundance. The red arrows indicate observed cleavage sites.

We further analyzed the status of protein N-termini by counting the number of PSMs and by calculation of the amino acid frequencies of the terminal residues (Fig. 4). The dbTIS dataset, except the ‘non-terminal residue category’ (2,730 N-termini), showed that the amino acid preference was different between acetylated and free protein N-termini, and also between removed and retained iMet. The degree of acetylation appeared similar between removed and retained iMet (Fig. 4A). We found a high prevalence of alanine and serine at the P1′ position of N-termini without acetylated iMet, whereas the preferred amino acids for free protein N-termini were proline, alanine, valine, and glycine, respectively. In the case of N-termini with iMet, acetylated iMet was mostly followed by large polar residues (glutamate, aspartate). In addition, lysine was the major residue that followed free iMet (Fig. 4B). Several studies have previously investigated Nt-acetylation in human cell line^29^, mouse skin^30^, and human platelets^27^. The amino acid preference of our dbTIS acetylated protein N-termini is consistent with these studies. It is worth noting that proline, valine, and glycine in the N-termini were rarely acetylated, as also observed in separate *Drosophila melanogaster* studies^31^. According to the study, a proline sitting at N-terminus or the second position prevents acetylation reaction of Nα-terminal acetyltransferases (NATs).

Nt-acetylation generally occurs co-translationally by NATs with acetyl-coenzyme A during protein synthesis. In humans, various NATs are expressed such as hNatA, hNatB, hNatC, hNatD, hNatE and hNatF^5, 32, 33^. These NATs differ in substrate specificity, and each NAT acetylates at one or more N-terminal amino acid sequences. In particular, NatA acetylates N-termini after iMet is removed by methionine aminopeptidase. It also acetylates Asp- and Glu- N-termini of mature actins post-translationally. Table 1 shows the counts of N-termini for each NAT. Out of 2,172 acetylated protein N-termini, 1,986 were mapped as the substrates of NATs, and 67% of the mapped N-termini were predicted as NatA substrates. The substrates of NATs were identified as acetylated and free forms. Thus, we determined the relative level of Nt-acetylation based on the number of PSMs of each substrate. The relative level of Nt-acetylation for NatA substrates was 76.7% in average. It was 92.8% for NatB, 72.2% in NatD and 45.3% in NatC/E/F. The results showed that most of the NAT substrates were present in the state of acetylation. However, the acetylation efficiency was different between NATs and even between substrates of each NAT. Gly and Val among NatA substrates and ML, MW, MK and MA among NatC/E/F substrates were detected less acetylated than other substrates. Interestingly, protein N-termini starting with Gly and Val were observed mostly free compared to other substrates of NatA. In addition, other papers published previously showed similar results^31, 34, 35^. While there appears to be little knowledge about the efficiency of NatA on each of its substrates, it is clear that the acetylation efficiencies at Gly and Val are much lower than the acetylation at other substrates of NatA.

**Table 1:** Classification of dbTIS^1^ protein N-termini according to the substrate type of N-α-terminal acetyltransferases in human (NATs).

Type of NAT	Substrates	# of protein N-termini		# of PSM^2^		Acetylated (%)
		Acetylated	Free	Acetylated	Free	
NatA	A	875	67	52358	9212	85.0
	C	7	1	935	20	97.9
	G	49	60	932	6441	12.6
	S	299	11	12967	951	93.2
	T	63	9	4375	1147	79.2
	V	34	63	1706	6570	20.6
	D	1	0	4942	257	95.1
	E	1	0	3861	301	92.8
	Total	1329	211	82076	24899	76.7
NatB	MD	138	1	9206	567	94.2
	ME	175	4	8877	811	91.6
	MN	78	5	2623	230	91.9
	Total	391	10	20706	1608	92.8
NatD	S	3	0	432	166	72.2
NatC/E/F	MI	15	6	1053	870	54.8
	ML	64	14	1525	2063	42.5
	MF	37	9	1351	293	82.2
	MW	6	4	40	103	28.0
	MK	41	49	762	8743	8.0
	MA	44	28	357	473	43.0
	MM	16	1	3051	145	95.5
	MV	40	16	3680	1599	69.7
	Total	263	127	11819	14289	45.3
Others –iMet^3^ removed	E	9	7	30	122	19.7
	I	1	0	1	0	100.0
	K	1	1	20	1	95.2
	M	7	5	755	146	83.8
	N	4	0	5	1	83.3
	P	41	129	4826	21029	18.7
	Q	1	3	1	3	25.0
	D	0	2	0	5	0
	R	0	1	0	5	0
	Y	0	1	0	1	0
	Total	64	149	5638	21313	20.9
Others –iMet retained	MC	0	1	0	3	0
	MG	8	4	27	92	22.7
	MH	6	2	245	125	66.2
	MP	19	10	620	954	39.4
	MQ	34	4	2013	3942	33.8
	MR	11	15	96	263	26.7
	MS	20	15	145	91	61.4
	MT	21	4	674	266	71.7
	MY	3	6	19	12	61.3
	Total	122	61	3839	5748	40.0

^1^dbTIS: UniProtKB-annotated translation initiation sites, ^2^PSM: Peptide spectrum match, ^3^iMet: initiator Met.

Next, we looked for cleavage sites of signal/transit peptides and propeptides. Most of the N-termini exposed by removal of the signal/transit peptide or propeptide were observed to be in non-acetylated states. Visualized patterns in the peptide sequences by generating an iceLogo^36^ for the 30 residues between P15 and P15′. The results show the strongest enrichment for arginine residues at P3 and P2 positions of the transit peptide and leucine residues at the P15-P6 position of the signal peptide (Fig. 4C), as likely observed from previous studies^23^.

### Identification of alternative translation initiation sites

**Figure 5:**
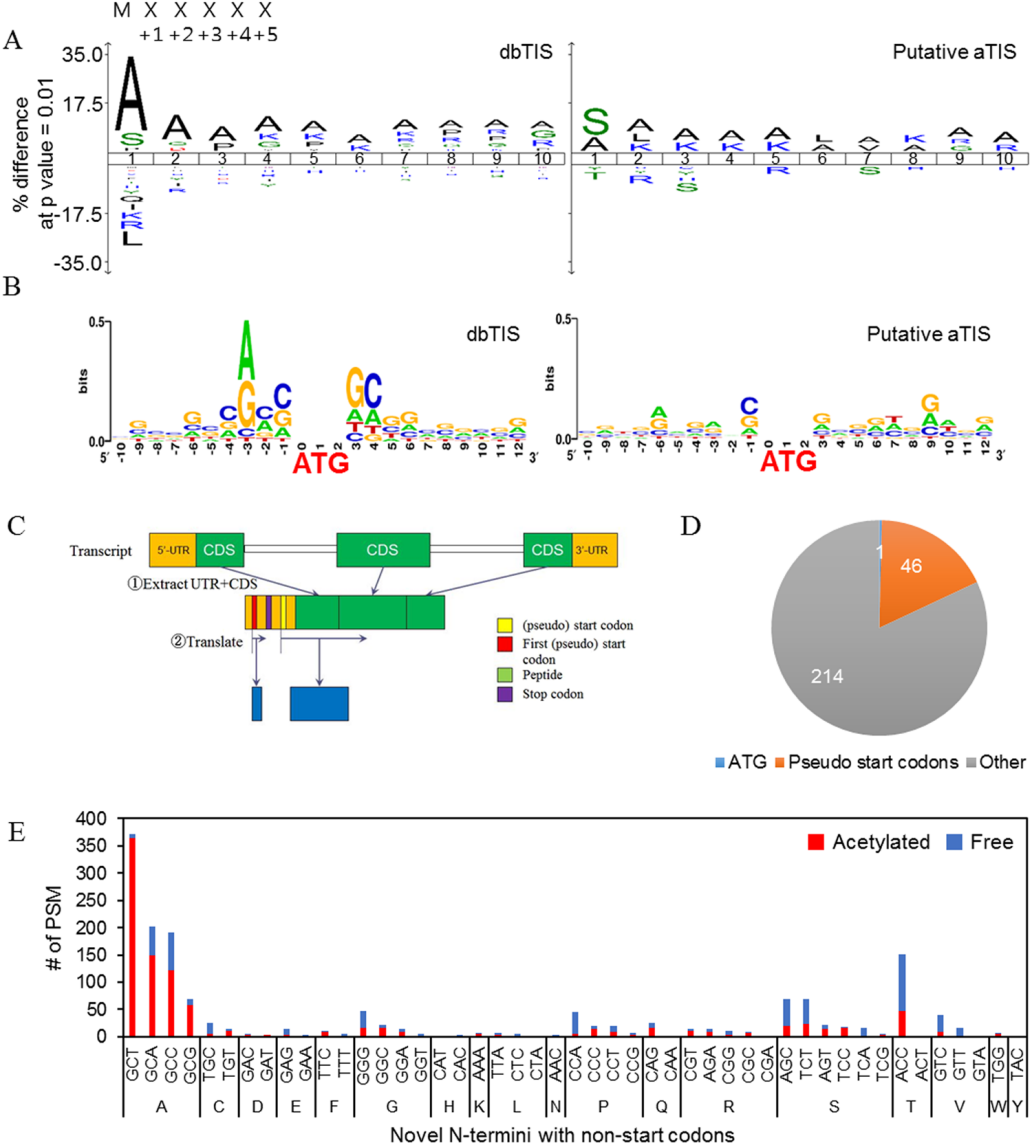
Putative alternative translational initiation sites. (**A**) iceLogo diagrams for amino-acid occurrences between dbTIS and putative aTIS. The amino acid frequencies after any methionine (either iMet or internal Met) in the human Swiss-Prot database (release 2015. 1) were determined for use as background correction. The sequences start immediately after methionine. (**B**) Nucleotide sequences at the flanking region of the initiator methionine residue. The central ATG is the codon for the initiator methionine of dbTIS (left) and putative aTIS (right). (**C**) The design of NtermDB. It is designed to allow a search for novel protein N-termini within an upstream UTR region. Orange blocks represent UTR regions, and green blocks represent coding sequence regions (CDS). Novel protein N-terminus was assumed to start at the start codon (“ATG”) or a pseudo start codon along the same frame as that of the matching CDS. We chose the farthest upstream (pseudo) start site and in-silico translated the transcript model. See methods for more details. (**D**) Codon usage in the identified novel N-termini. Nucleotide sequences corresponding to the first residue of the identified 5′-UTR peptides are presented. (**E**) Number of PSMs for acetylated or free N-terminal 5′-UTR peptides starting with non-start codons.

In eukaryotes, ribosomal translation initiation may occur alternatively at upstream of the annotated coding sequence or downstream of in-frame ATG codons^37, 38^. Ribosome profiling is a useful tool that is based on sequencing potential translation start sites of mRNA^39, 40^. In order to discover putative aTIS at protein level, we analyzed our MS data using not only UniProtKB database but also a customized database containing in-silico-translated 5′-UTRs. Especially for 5′-UTR translation, we were interested in finding of proteomic clues for the possible expression of genes from pseudo start codons. First of all, 495 of unannotated protein N-termini were inferred to be putative aTIS downstream of the canonical start site. These included protein N-termini starting with internal methionine no matter they are acetylated (223) or free (272) and no matter the methionine was retained (234) or removed (261). We used iceLogo to compare the amino acid frequencies after iMet at dbTIS and at putative aTIS using all amino acid sequences after any Met in the human Swiss-Prot database to establish background amino acid frequencies. Both of the logos showed high preferences for alanine and serine at the position immediately after iMet and subsequent preferences for mainly alanine (Fig. 5A). In the putative aTIS category, the two amino acids were identified with a total frequency of 32% at the first position following iMet (Supplementary Fig. S2). The nucleotide consensus sequences that surrounded the ATG codon of dbTIS and putative aTIS showed a similar pattern of Kozak sequences^41–43^ (Fig. 5B). On the other hand, 77 out of 495 putative aTIS were mapped to the uppermost iMet of alternative splicing transcripts in the ECgene alternative splicing database (Supplementary Table S3)^44^, implying that these putative aTIS may not be true aTIS, but act as canonical TIS in case of an alternative splicing event generating shorter transcripts. Given these interpretations, it appears that the protein N-termini in our putative aTIS category originated from alternative translation initiation or from translation after alternative splicing.

While aTIS at downstream of dbTIS can be identified using the UniProtKB database, those events starting at upstream of dbTIS cannot be discovered using the current reference protein databases. Therefore, we constructed a novel protein sequence database, ‘NtermDB’ (detailed in Methods), which included in-silico-translated sequences of 5′-UTRs of known coding sequence (CDS) regions from the site of a start codon (ATG) or its single-nucleotide variants, pseudo start codons (CTG, TTG, GTG, AGG, ACG, AAG, ATC, ATA and ATT) (Fig. 5C). These putative aTISs were mapped to 67% of the total transcripts in the Ensembl database (http://www.ensembl.org/index.html). The sites were evenly distributed among 23 chromosomes, with the exception of chromosome Y. MS/MS spectra unmatched in the first search using UniProtKB database were subsequently searched against the NtermDB. As a result, we identified 261 novel protein N-termini (from 394 transcripts). Of these transcripts, five transcripts were found to be identical to those of Ribo-seq data of Lee, S. *et al*.^40^. All of these were transcribed at the 5′-UTR using the pseudo start codons (Supplementary Table S4). The protein N-termini were found on all chromosomes, with the exception of the Y chromosome and chromosome 21. Moreover, the protein N-termini were mostly distributed on chromosome 1 (Supplementary Fig. S3a). Of the 261 novel protein N-termini, 46 was identified in the 5′-UTR with an acetylated or free pseudo start codon (Fig. 5D; Supplementary Fig. S3b; Supplementary Table S4). The remaining 214 protein N-termini were found with other (pseudo) start codon, more precisely, the N-termini were found to be associated with four codons i.e., GCT, GCA, GCC and GCG, which code alanine (46 termini; Fig. 5E, Supplementary Fig. S3c, Supplementary Table S4). Most of such protein N-termini starting with alanine were identified as acetylated and with only one exception there was no intervening stop codon until the beginning of CDS. Of the 46 N-termini starting with alanine, 72% were identified with peptides overlapping the canonical CDS region. Therefore, we are confident that we have identified novel N-termini. Furthermore, 39% had pseudo start codon prior to alanine. In most of the cases like the current study and other previous investigations when iMet is followed by alanine, iMet is cleaved and then newly exposed alanine is acetylated. For this reason, it is highly likely that the protein N-termini which have an alanine at the first residue and pseudo start codon at its preceding residue are alternative translation initiation site in 5′-UTR.

### Unknown processing sites

**Figure 6:**
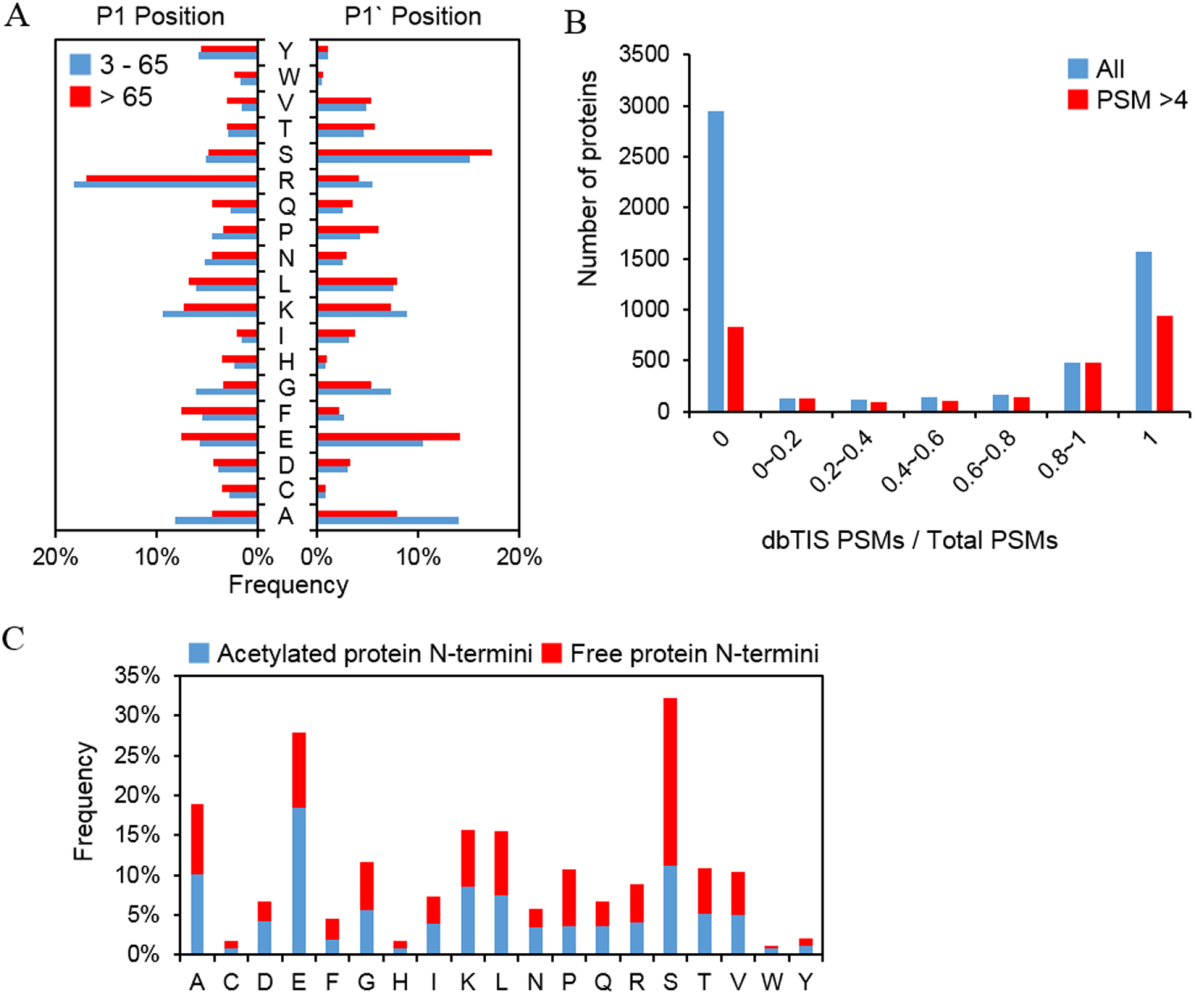
Characterization of protein N-termini from the category of unknown processing. (**A**) Amino acid distributions at P1 and P1′ positions of protein N-termini identified at residue 3–65 (blue) and >65 (red) along the protein sequences. (**B**) A number of proteins according to the proportion of dbTIS PSMs. ‘1’ for x-axis value means that all PSMs are matched to dbTIS, ‘0’ means that the protein was identified only with PSMs corresponding to unknown processing category, and the intermediate values mean that the protein was identified with both type of PSMs. (**C**) Distribution of acetylated and free protein N-termini belonging to unknown processing category.

Out of 10,103 protein N-termini, only 5% are predicted as putative aTIS, and the remaining 95% are derived from hitherto unknown processing. We were very concerned about finding a way to distinguish between free N-termini in cells and internal sites exposed during sample processing in order to ascribe features to the unannotated N-termini. Chemical labeling of amine groups at the protein level is required to discriminate not only between endogenous Nt-acetylated N-termini and endogenous free N-termini but also between the N-terminal peptide and internal peptides of protein. However, if the inactivation of the N-blocking reagent is not complete, it is possible that the α-amine of the internal peptide on N-terminal residue is labeled by trace N-blocking reagent remaining after or during digestion and these internal peptides could be falsely identified as protein N-termini. Therefore, during the experiment, we performed an inactivation step of the chemical label with hydroxylamine followed by FASP digestion, and after MS database search, we picked out protein N-termini without a protease-specific site at the N-terminal end of the identified peptide. Hydroxylamine has also a beneficial effect to revert unwanted O-acylation that might happen during the labeling reaction^13^. Interestingly, almost half of the resulting protein N-termini of unknown processing category (43%) were identified as acetylated forms. Most unannotated protein N-termini were also identified in eukaryotes, no matter whether researchers used negative^17, 20^ or positive^23^ enrichment methods. If we compared our dataset to the database generated by Crawford *et al*. (‘Degrabase’), focusing on free protein N-termini at positions 3–65, we observed a high similarity of the patterns of amino acid frequencies (Supplementary Fig. S4). Thus, it appears likely to us that the free protein N-termini are newly-exposed N-terminal sites obtained after removal of putative signals or transit peptides (according to the an interpretation by Crawford *et al*.). Aminopeptidase ragging events might alter initially generated cleavage products. When we compared protein N-termini at positions >65 to those at positions 3–65, the data showed a similar amino acid distribution at the P1 and P1′ positions (Fig. 6A), with predominance of arginine at P1. From the results, we suspect that cleavage of signal/transit peptide and degradation of proteins is mainly caused by trypsin-like.

Protein N-termini for a total of 5,727 proteins were characterized in our study. Of them, 2,591 proteins retained intact N-terminal sequence (dbTIS), whereas 3,968 proteins were found in truncated forms with no known signal/transit or propeptide sequence. Such truncated proteins have also been reported mainly by the TAILS method. It is noteworthy that 83% of 5,537 proteins in the two categories were found with either intact (28%, ‘1’ category) or truncated (53%, ‘0’ category) sequences, but not both features (Fig. 6B). This suggests that protein degradation is not the main cause of truncation. Rather, such truncation seems to have an influence on functional diversity in the human proteome, and a considerable amount of protein N-termini in the unknown processing category may represent mature physiological forms in HEK293T cell.

We also observed the frequency of N-terminal residues between acetylated and free protein N-termini (Fig. 6C). There was a preference for glutamate and aspartate in acetylated protein N-termini and serine and proline in free protein N-termini. Protein degradation in eukaryotic cells is mainly carried out by the ubiquitin/proteasome system and regulated by the N-end rule. Recently, Lange *et al*. suggested a modified N-end rule based on the observed amino acid frequency and Nt-acetylation status of internal protein N-termini in human erythrocytes^20^. Glutamate and aspartate were classified as “acetylation-stabilized” or “free non-destabilizing,” whereas serine was classified as “acetylation destabilized.” The two acidic residues are defined as secondary residues by the Arg/N-end rule. Thus, acetylation at N-terminal glutamate and aspartate would inhibit the destabilization of arginylation, which in turn increase the stability of proteins. In contrast, proline was observed to be free at the N-termini, reflecting the well-known fact that proline structurally interferes with acetylation and make free N-terminus. Thus, these different amino acid sequences seen in the protein N-termini of unknown processing category appear to be derived from protein stability by the Nt-acetylated state.

N-terminal acetylation occurs co-translationally by NATs, however, while the exact mechanism for post-translational Nt-acetylation is unknown yet, a feature of post-translational Nt-acetylation in yeast has been reported^26^. We identified 4,088 acetylated protein N-termini at >2 position (Fig. 4A). Of these, approximately 50% have trypsin or GluC specific terminals. We controlled acylation in the course of the experiment, so we do not know the exact cause of this phenomenon. One possible explanation is the misannotation between the N-terminal acetylation and the ε-acetylation of internal lysine since we forcibly set acylation at all ε-amine of lysine residues during database search. Except for the N-termini with enzyme specific sites, glutamate was mainly observed in P1` position (Supplementary Fig. S5). There is a report that acetylated mature actins harboring Asp- or Glu- on N-termini are produced by post-translational modificatio^45^. Although our result is seen as affected by each enzyme in P1 position, it is still possible that the acetylation was regulated by post-translational modification. To assess our unknown processed N-terminome data further, we exploited the information from a set of published data^29^ and from the TopFIND database (http://clipserve.clip.ubc.ca/topfind/). In the case of Nt-acetylation at dbTIS, most of the protein N-termini, regardless of the type of samples and experimental methods, were identified in the human proteome. More than 50% of the identified dbTIS overlapped between any two sets of data (Supplementary Fig. S6a). In contrast to this protein N-termini at position 1 or 2, there was a significant disparity between the data of protein N-termini at positions >2. In the paper using COFRADIC^16^, the information of acetylated protein N-termini at positions >2 was not available and could not be compared, while a considerable number of protein N-termini were reported by a paper using TAILS method^25^ (Supplementary Fig. S6a). The protein N-termini at positions >2 in the dental pulp proteome^17^ were identified in similar proportions in our study. However, very few protein N-termini at position >2 were commonly found in both studies (Supplementary Fig. S6b). Interestingly, ontology analysis revealed that the “large ribosomal subunit,” “melanosome,” “nuclear chromosome” and “mitochondrial part” were mostly enriched in protein N-termini of positions >2 (Supplementary Fig. S7). Therefore, we infer that the post-translational Nt-acetylation is more affected by the type or status of the sample than co-translational Nt-acetylation.

## Conclusion

Our characterization of protein N-termini offers information of overall protein status in cells. We identified 13,095 protein N-termini from 5,727 proteins with FDRs ≤0.01. Use of multiple N-blocking reagents and proteases led to an increase in the N-terminome coverage (Fig. 2). The study proposes an approach for the enrichment of protein N-termini to assess protein status, including Nt-acetylation status, signal/transit peptide removal and truncation of protein (Fig. 3). Moreover, it provides not only the putative aTIS, but also N-terminal peptides mapping to the 5′-UTR or downstream to the in-frame ATG codons supposed to be alternative TIS (Fig. 5). We have also observed the features of post-translational Nt-acetylation that were not previously known and discovered a large number of proteins in truncated forms (Fig. 6). By combining two different N-blocking reagents, two endoproteases, three search engines and two databases, Nrich could accelerate and expand the high-throughput identification of the N-terminome to arrive at an accurate identification of the entire human proteome under different conditions *in vitro* and *in vivo*.

## Methods

### Materials

Sequencing-grade modified trypsin was purchased from Promega (Madison, WI, USA), endoproteinase Glu-C from Sigma-Aldrich (St. Louis, MO, USA), Tris(2-carboxyethyl)phosphine hydrochloride (TCEP-HCl) and NHS-Activated Agarose Slurry from ThermoFisher Scientific (Rockford, IL, USA), S-methyl methanethiosulfonate (MMTS) from TCI (Tokyo Chemical Industry, Tokyo, Japan), and C18 MacroSpin Column from NestGroup (Southborough, MA, USA).

### Cell culture and protein extraction

HEK293T cells were grown in DMEM (Gibco, Rockville, MD, USA) medium supplemented with 10% FBS (Gibco) and 1% penicillin/streptomycin (Gibco). Cultures were maintained in an atmosphere of 5% CO_2_ and 95% air in a humidified incubator at 37 °C. Cells were grown to 70–80% confluence. Before harvesting, cells were washed twice with ice cold phosphate buffered saline (PBS, Gibco®). The HEK293T cells were harvested by centrifugation and suspended in ice cold lysis buffer containing 6 M guanidine and 0.1 M HEPES (pH 8.5). At this step, we did not include any other proteases because the absence of inhibitors did not affect the results of our downstream experiments (Supplementary Fig. S1). After 10 min incubation on ice, cells were lysed by ultrasonication. The proteins from the cell lysate were isolated by transferring supernatant after centrifugation at 13,200 × *g* for 10 min at 4 °C. The protein concentration of the collected supernatant was determined by BCA (bicinchoninic acid) protein assay (ThermoFisher Scientific).

### Enrichment of N-termini

The proteins extracted from HEK293T (5 mg, 1 mg/mL in lysis buffer) were reduced with 5 mM TCEP for 2 hours and alkylated with 15 mM of MMTS for 1 hour. Thereafter, the pH of the protein sample was shifted to 12 by titrating with 6 M NaOH. Labeling of amine group was carried out by treatment of 150 mM of propionic anhydride (Sigma-Aldrich) or D6-acetic anhydride (Cambridge Isotope Laboratories, Tewksbury, MA, USA) for 1 hour at 25 °C. To achieve complete amine labeling, the labeling step was repeated one more time. Unreacted anhydrides was quenched with 20 mM of hydroxylamine for 30 min. All reactions were performed at 25 °C with gentle shaking at 600 rpm. After labeling reaction, the sample was transferred onto Amicon® Ultra-15 30 K molecular weight cut-off filter (Millipore, Billerica, MA, USA) pre-activated by 0.1 M HEPES (pH 8.5). Proteins on top of the filter were washed with 10 mL of 50 mM ammonium bicarbonate and then 20 mL of 0.1 M HEPES (pH 8.5). Subsequently, the proteins were digested by treatment of endoprotease (trypsin or GluC at 1:50 enzyme:protein ratio) for 16 hours at 37 °C. Finally, digested peptides were eluted from the filter twice with 5 mL of 0.5 M NaCl, 0.1 M HEPES (pH 8.5) at 3000 × *g* for 5 min.

The peptide solution was transferred into NHS-activated agarose slurry dissolved in 0.5 M NaCl, 0.1 M HEPES (pH 8.5). The peptide-slurry mixture was incubated with gentle up-and-down rotation for 2 hours at 25 °C. After incubation unbound fraction was separated using 0.22 μm cellulose acetate filter (Corning, New York, NY) followed by a wash with 50 mL of 0.5 M NaCl, 0.1 M HEPES (pH 8.5). The peptides in the flow-through were purified using MacroSpin Spin Column (300 μg, NestGroup). About 8 mL of sample was loaded onto a column by repetitive binding-and-centrifugation and washed with 2 mL of 10 mM ammonium formate (pH 10). Elution of peptides was done by stepwise increment of acetonitrile composition in 10 mM ammonium formate (pH 10) elution buffer: 10%, 22.5% and 35% acetonitrile eluates were combined into one fraction, 12.5%, 25% and 37.5% were into another, 15%, 27.5% and 40% were into the third, 17.5%, 30% and 42.5% were into the fourth, 20%, 32.5% and 45% were into the fifth and 80% acetonitrile eluate was into the last sixth fraction. The six peptide fractions were dried in vacuo and kept at −80 °C until use.

### Liquid chromatography and tandem mass spectrometry

Dried peptide samples were reconstituted in 0.4% acetic acid, and an aliquot containing approximately 1 μg was injected from a cooled (10 °C) autosampler into a reversed-phase Magic C18aq (Michrom BioResources, Auburn, CA, USA) column (15 cm × 75 μm, packed in-house) on an Eksigent nanoLC-ultra 1D plus system at a flow rate of 300 nL/min. Prior to use, the column was equilibrated with 90% buffer A (0.1% formic acid in water) and 10% buffer B (0.1% formic acid in acetonitrile). The peptides were eluted with a linear gradient from 10% to 50% buffer B over 120 min and 50% to 80% buffer B over 5 min followed by an organic wash and aqueous re-equilibration at a flow rate of 300 nL/min with a total run time of 150 minutes. The HPLC system was coupled to a Q-Exactive mass spectrometer (ThermoFisher Scientific, Bremen, Germany) operated in a data-dependent acquisition (DDA) mode. Survey full-scan MS spectra (m/z 300–1800) were acquired with a resolution of 70,000. Source ionization parameters were as follow: spray voltage, 2.5 kV; capillary temperature, 300 °C; and s-lens level, 44.0. The MS/MS spectra of the 12 most intense ions from the MS1 scan with a charge state 1~5 were acquired with the following options: resolution, 17,500; automatic gain control (AGC) target, 1E5; isolation width, 2.0 m/z; normalized collision energy, 27%; dynamic exclusion duration, 90 s; and ion selection threshold, 4.00E + 03 counts.

### Conventional database search

The RAW-files from the Q-Exactive were converted into mgf-files using the Proteome Discoverer version 1.4 software (ThermoFisher Scientific) with default options. All spectra were first matched to peptide sequence in the UniProtKB human database (release 2015. 1) using MS-GF +(v10072)^46, 47^ and Comet (2016.01 rev. 2)^48^ search engines. The search engine settings were as follow: 1 as the number of tolerable termini; 15 ppm for MS1 tolerance; 0, 2 as IsotopeErrorRanges; allow to decoy database search; variable modifications: oxidation of methionine (+15.9949 Da), acetyl (+42.0106 Da) or chemical label (Acetyl:2 H(3) or propionyl) of N-term; fixed modification: methylthio of cysteine (+45.9877 Da) and chemical label (Acetyl:2 H(3) or propionyl) of lysine. Search outputs from MS-GF+ and Comet were conveyed to Percolator^49, 50^ for estimation of PSM (peptide spectrum match) level FDR of peptide identification. The false discovery rate (FDR) was set to 1% at the PSMs level. Subsequently, the unidentified spectra from the two search engines were re-analyzed by the MOD^i^ algorithm^51^. The search parameters were the same as for the other two search engines. FDR for MOD^i^ results was estimated using the standard Target-Decoy method and the PSMs at 1% FDR were collected. All peptides identified in any one search engine were collected. If MS-GF+ and Comet outputted different PSMs for a single spectrum, the PSM with higher q-value was discarded.

### Database for novel protein N-termini

While novel protein N-terminus starting at downstream translation initiation site can be identified using the reference protein database, those starting at upstream translation initiation sites cannot be discovered. We constructed a novel protein sequence database, “NtermDB,” which includes translated sequences starting at upstream TIS, possibly extending into some parts of known coding sequence (CDS) region. The design of NtermDB is based on a few assumptions and design goals.

In recent reports by Global mapping of translation initiation sites in mammalian cells at single-nucleotide resolution^40^, it was shown that codons at these potential upstream TIS were mostly either the START codon ‘ATG’ or its single nucleotide variants. For each transcript model in the reference genome DB, we generated translated sequences that included potential novel protein N-terminus. During the translation, we made an assumption that the codons at the novel upstream TIS can only be either the START codon or pseudo start codons, i.e., SNVs of the ATG start codon. We also assumed that the translation frame of the CDS region for a given transcript is consistent with the novel upstream TIS. In a 5′-UTR of a gene, there may be many (pseudo) start codons. If each of these (pseudo) start codons is assumed to be the start site of a potential novel protein N-terminus, there will be many redundant translated sequences in NtermDB. Thus, we choose the (pseudo) start codon position that is farthest upstream from the reference start site as the potential upstream TIS and generate a single translated sequence from a transcript model unless the translation is stopped by a STOP codon. When a STOP codon is met, translation is stopped, and we skip nucleotide sequences until another (pseudo) start codon is met within the 5′-UTR and start generating another translated sequence (Fig. 5C). Translation of a 5′-UTR is extended into a CDS region up to a maximum of 50 amino acids or by allowing up to five miscleavages, downwards from the reference start site so that the redundancy between NtermDB and the reference DB is minimized while any peptide spanning over 5′-UTR and CDS can be identified. We used Ensembl Release 78 for transcriptome model, UniProtKB Database 2015 January release version, and the mapping table between the two downloaded from the UniProt website (http://www.uniprot.org/).

All of the MS/MS scans that were identified with scores above the 1% FDR threshold in the first round of conventional search was removed from the input spectra. The remaining unidentified spectra were submitted to the second stage search for novel protein N-termini. For this, the same three search engines were used against NtermDB at the same parameter settings as the first-round search. We applied this multi-stage search strategy because it is reported that the method is more conservative on the FDR estimation for novel peptide identification when compared with single stage search^52^.

### Other bioinformatic analyses

Sequence logos were made using iceLogo (http://iomics.ugent.be/icelogoserver/index.html) and Weblogo (http://weblogo.berkeley.edu/logo.cgi). All logo images were made with the percent-difference scoring system. Data for methionine processing, transit peptide removal, and signal peptide removal were compared with the Swiss-Prot library release 2015.1. Terminus (http://terminus.unige.ch/) was used to predict initial methionine cleavage and acetylation of protein N-termini.

### Accession number

The mass spectral dataset (PXD0055831) representing the proteomics data has been deposited with the ProteomeXchange Consortium (http://proteomecentral.proteomexchange.org) via the PRIDE partner repository.

## Electronic supplementary material

Supplementary Information

Supplementary tables
