# Supplementary material for: Comprehensive analysis of human protein N-termini enables assessment of various protein forms
Source: Sci Rep. 2017 Jul 26;7:6599. doi: 10.1038/s41598-017-06314-9 (PMC5529458; doi:10.1038/s41598-017-06314-9)
Supplement: Supplementary file 1 — Supplementary Information [file 41598_2017_6314_MOESM1_ESM.pdf]

Supplementary Information

Comprehensive analysis of human protein N-termini enables assessment of various protein forms

Jeonghun Yeom[1,2], Shinyeong Ju[1,3], YunJin Choi[4], Eunok Paek[4], Cheolju Lee[*,1,2]

[1]Center for Theragnosis, Korea Institute of Science and Technology, Seoul 02792, Republic of Korea

[2]Department of Biological Chemistry, Korea University of Science and Technology, Daejeon 34113 Republic of Korea

[3]Department of Life Science and Research Center for Natural Sciences, Hanyang University, Seoul 04763, Republic of Korea

[4]Department of Computer Science and Engineering, Hanyang University, Seoul, Republic of Korea

[*]Corresponding author: Cheolju Lee. Phone: +82-2-958-6788; E-mail: clee270@kist.re.kr.

Supplementary Figures

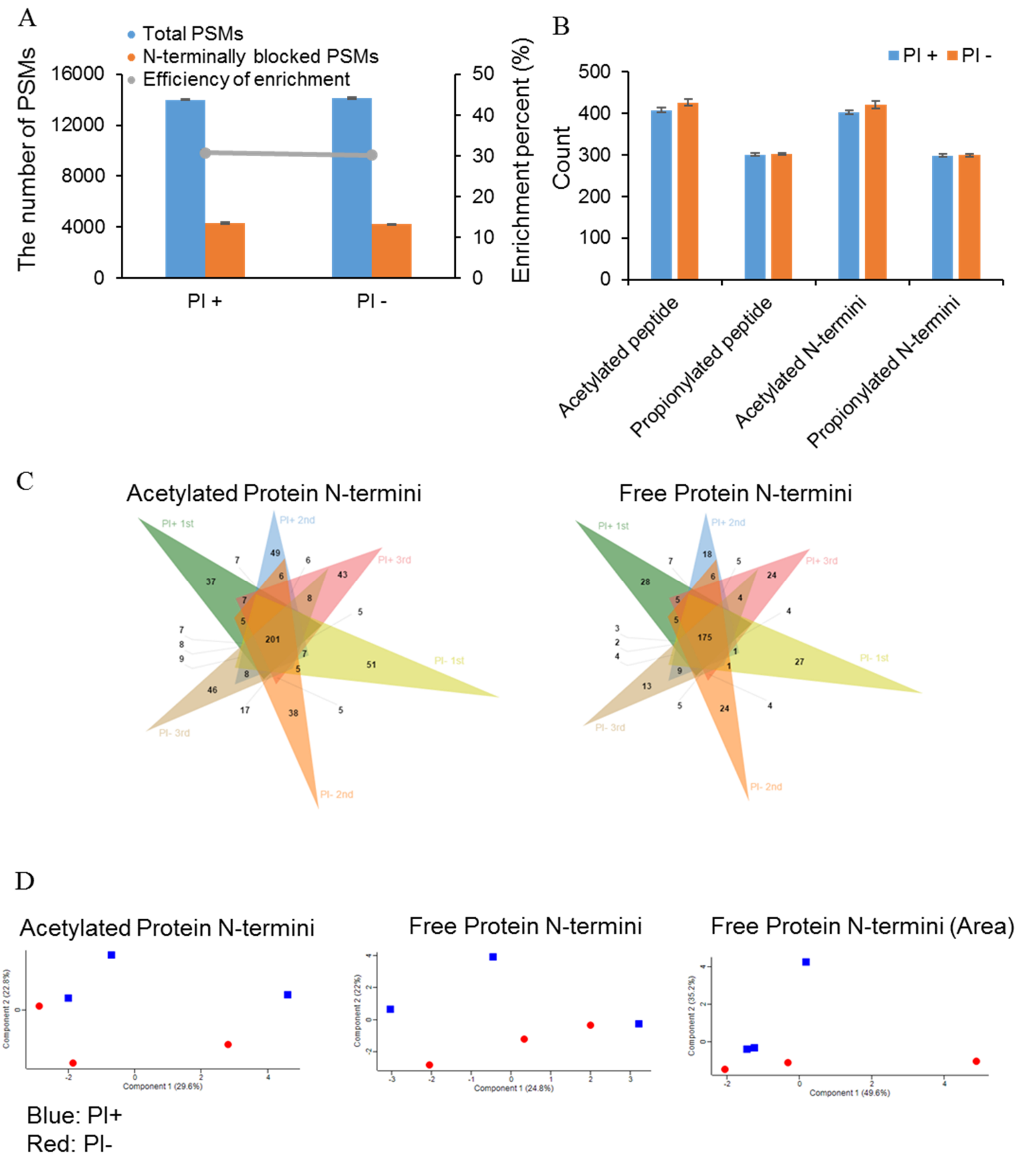

Figure S1. Comparison of the protein N-termini datasets prepared with or without protease inhibitors. We tested the effect of protease inhibitors using HEK293T lysate (200 µg). To confirm this, we used 6M guanidine alone in the lysis buffer (0.1M HEPES, pH 8.5) or added protease inhibitor (Thermo Scientific Halt™ Protease Inhibitor Single-Use Cocktail, EDTA, PMSF) in addition. The lysate was propionylated with propionic anhydride and then digested with trypsin using

FASP methods. Next, the digested peptides were incubated with NHS-activated agarose resin. The experiment was performed in biological triplicate. The plots show comparisons between the number of PSMs found (A), the enrichment efficiency (A), the number of N-termini (B) and the type of N-termini (C) according to the presence or absence of protease inhibitors. PCA scores plot does not show clustering of datasets according to the presence and absence of protease inhibitor (D). The figure is analyzed based on PSM, or the precursor ion area from Proteome discoverer 2.1 platform.

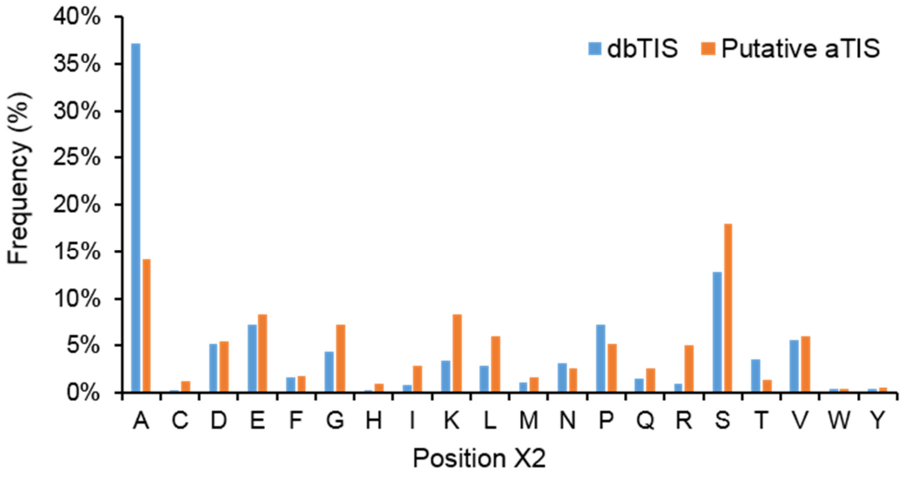

Figure S2. Features of putative alternative translational initiation sites. Amino acid frequencies are compared between dbTIS and Putative aTIS.

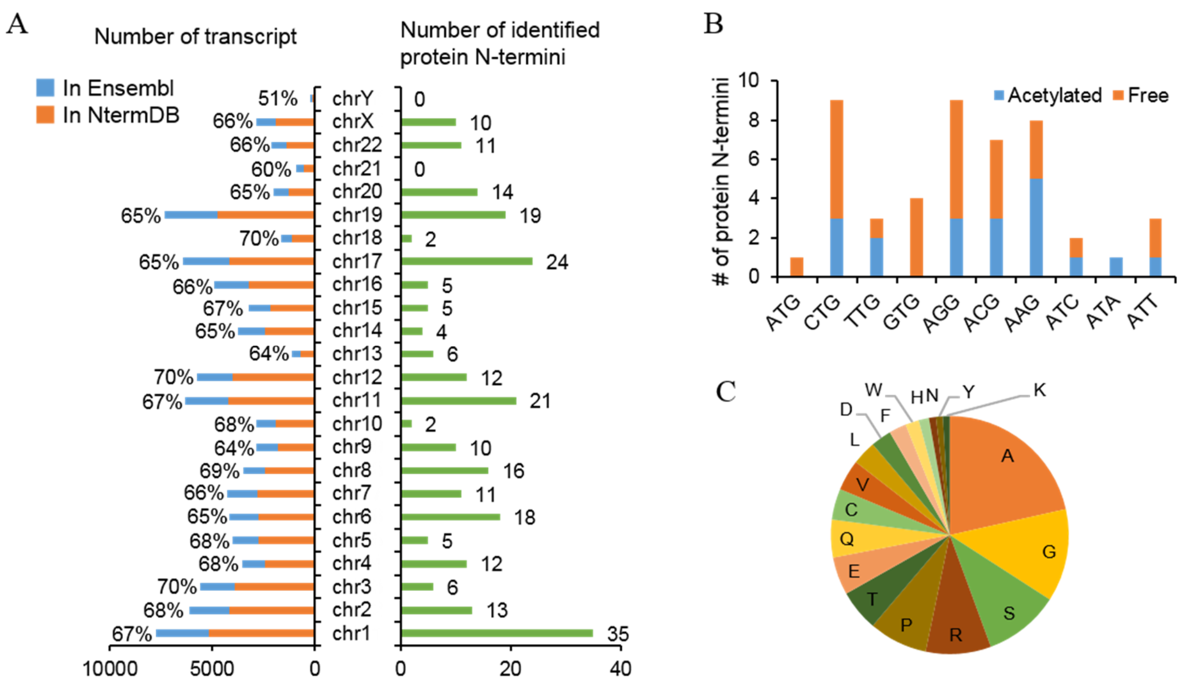

Figure S3. Features of novel protein N-termini discovered at 5′-UTR.

(A) Chromosomal distribution of the predicted and identified novel protein N-termini.

(B) A number of identified novel protein N-termini with ATG codon or pseudo start codons.

(C) Amino acid distribution of the first residue of novel protein N-termini.

AA	Our (1,340)								Degrabase (729)							
	P4	P3	P2	P1	P1'	P2'	P3'	P4'	P4	P3	P2	P1	P1'	P2'	P3'	P4'
-	65	31	0	0	0	0	0	0	39	14	0	0	0	0	0	0
A	156	141	144	108	171	167	175	176	99	91	97	80	146	109	83	98
C	17	20	18	57	9	19	6	9	9	15	12	15	0	0	1	0
D	23	34	28	41	28	28	53	50	24	9	11	33	6	14	28	42
E	43	59	61	46	85	44	50	63	36	27	28	9	11	23	38	73
F	34	29	38	113	42	53	31	36	19	13	21	43	23	42	21	14
G	112	117	100	98	111	116	129	106	59	42	48	35	101	38	89	69
H	19	22	25	44	15	33	23	17	11	11	11	9	11	33	7	10
I	40	42	36	21	40	64	51	52	17	24	14	2	11	31	21	16
K	97	99	102	77	108	137	148	145	41	47	48	130	40	28	36	23
L	95	96	121	100	98	165	132	131	62	49	65	56	35	74	52	49
M	56	53	53	0	0	25	13	15	20	33	28	35	36	17	13	8
N	35	30	28	98	26	40	38	49	14	14	14	26	13	13	20	22
P	104	50	83	47	67	41	83	101	52	31	47	26	1	12	63	77
Q	52	63	54	43	31	52	51	65	27	36	30	28	11	26	36	34
R	98	138	138	170	84	64	51	39	58	104	113	100	2	2	5	4
S	109	121	125	70	258	103	100	77	61	63	61	33	176	105	106	80
T	59	69	72	38	75	61	75	84	28	35	29	11	30	58	57	44
V	92	91	86	17	74	99	106	98	37	52	31	12	62	72	44	54
W	13	13	8	33	4	8	6	9	6	6	6	5	2	6	1	2
Y	21	22	20	119	14	21	19	18	10	13	15	41	12	26	8	10

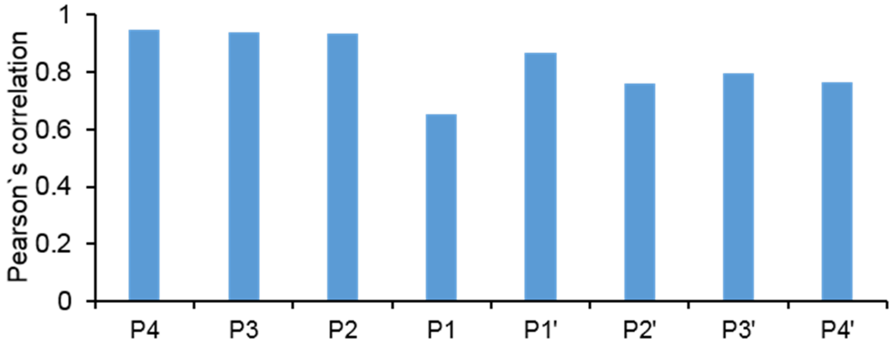

Figure S4. Comparison of the free protein N-termini at position 3-65 between our dataset and the database 'Degrabase' [21]. Amino acid frequencies are shown in a matrix where rows are amino acids and columns are positions. The graph at the bottom represents the correlation coefficient of amino acid frequencies between the two databases. The residue at P1′ is the N-terminal residue.

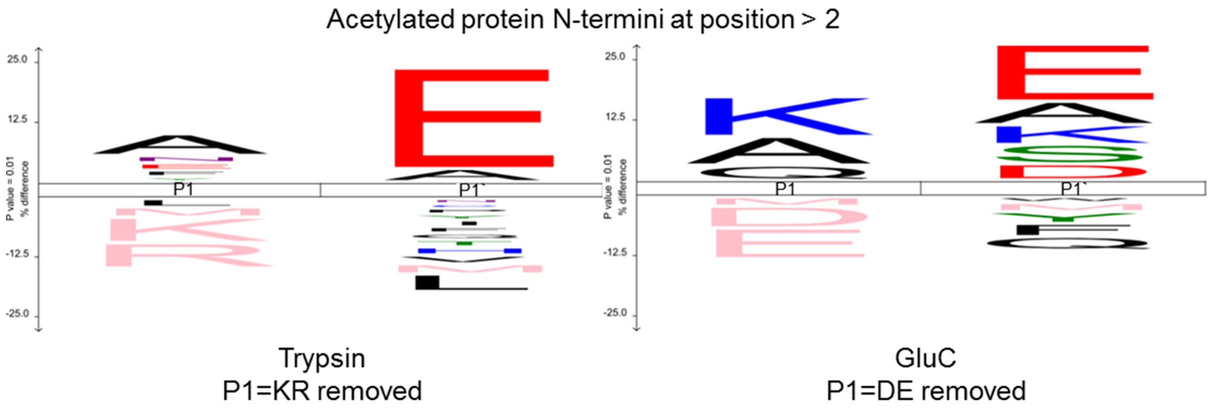

Figure S5. Comparison of the acetylated protein N-termini at position >2 between trypsin-experiment and GluC-experiment datasets. Then N-termini with enzyme specific sites at P1 were excluded from the analysis. The amino acid frequencies in the human Swiss-Prot database (release 2015. 1) were determined for use as background correction.

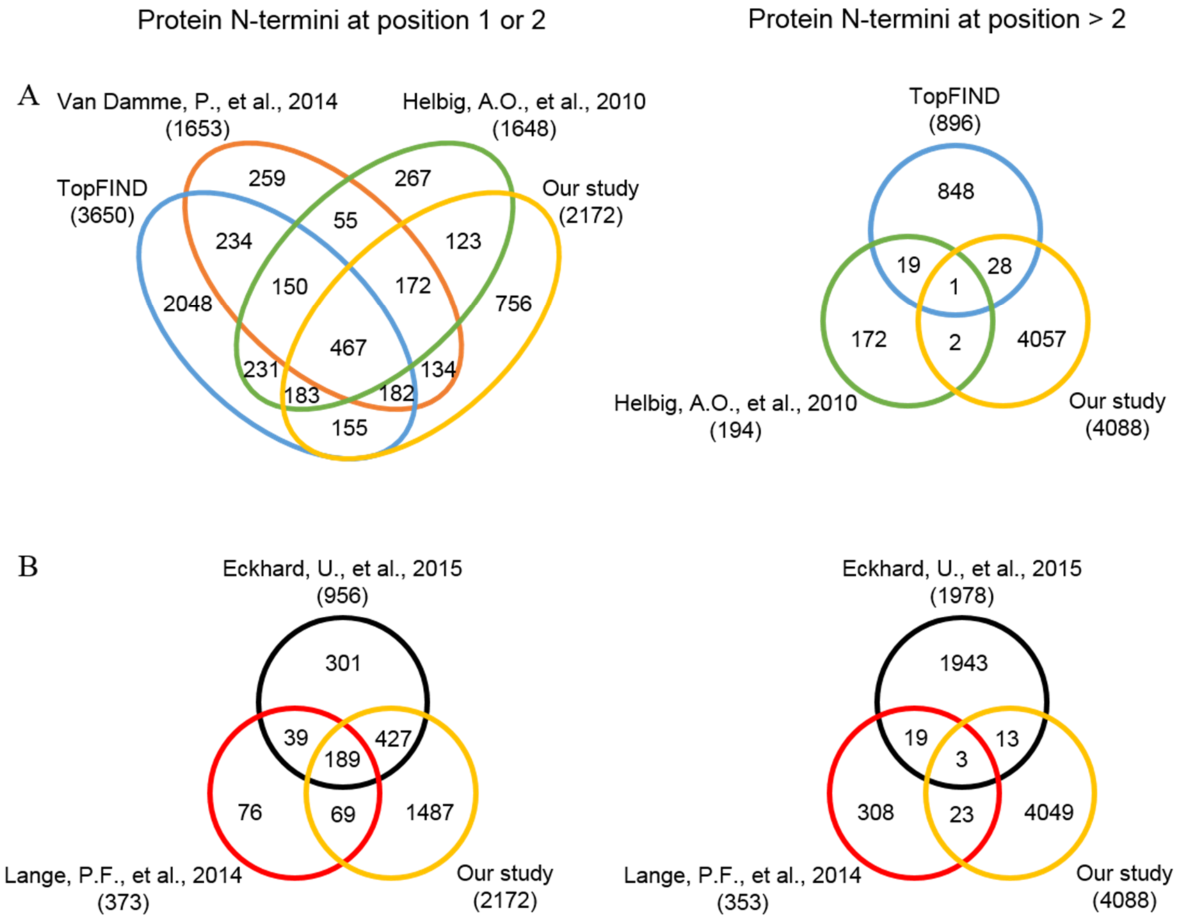

Figure S6. Comparison of the acetylated protein N-termini with previously-published N-terminome data studied with HeLa cervical carcinoma, HCT116 colorectal carcinoma, A-431 epidermoid carcinoma, THP-1 acute monocytic leukemia, K-562 chronic myelogenous leukemia, Jurkat leukemia, HEK293 kidney, and B-cells. (A), and with human erythrocytes and dental pulp (B).

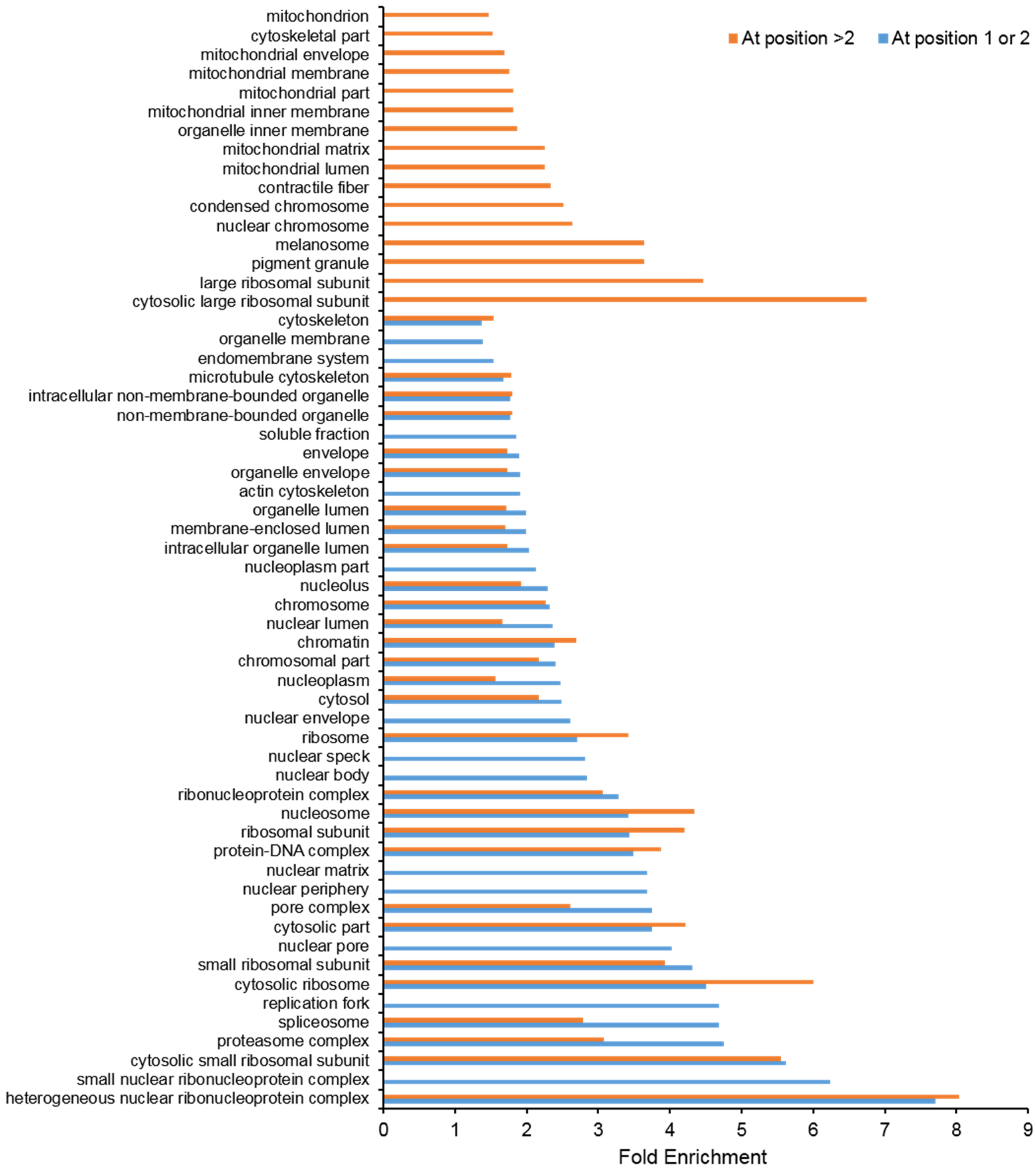

Figure S7. Gene ontology analysis of acetylated protein N-termini. The identified proteins are classified according to their localization (GO term: cellular component). The analysis was performed using the DAVID bioinformatics resources 6.7 (https://david.ncifcrf.gov/) with FDR < 0.01.
